# OGDD (*Olive Genetic Diversity Database*): a microsatellite markers' genotypes database of worldwide olive trees for cultivar identification and virgin olive oil traceability

**DOI:** 10.1093/database/bav090

**Published:** 2016-01-30

**Authors:** Rayda Ben Ayed, Hanen Ben Hassen, Karim Ennouri, Riadh Ben Marzoug, Ahmed Rebai

**Affiliations:** ^1^Centre of Biotechnology of Sfax, PB ‘1177’, 3018 Sfax, Tunisia and; ^2^Laboratory of Physics Mathematics and Applications, Faculty of Sciences of Sfax, Sfax, Tunisia

## Abstract

Olive (*Olea europaea*), whose importance is mainly due to nutritional and health features, is one of the most economically significant oil-producing trees in the Mediterranean region. Unfortunately, the increasing market demand towards virgin olive oil could often result in its adulteration with less expensive oils, which is a serious problem for the public and quality control evaluators of virgin olive oil. Therefore, to avoid frauds, olive cultivar identification and virgin olive oil authentication have become a major issue for the producers and consumers of quality control in the olive chain. Presently, genetic traceability using SSR is the cost effective and powerful marker technique that can be employed to resolve such problems. However, to identify an unknown monovarietal virgin olive oil cultivar, a reference system has become necessary. Thus, an Olive Genetic Diversity Database (OGDD) (http://www.bioinfo-cbs.org/ogdd/) is presented in this work. It is a genetic, morphologic and chemical database of worldwide olive tree and oil having a double function. In fact, besides being a reference system generated for the identification of unkown olive or virgin olive oil cultivars based on their microsatellite allele size(s), it provides users additional morphological and chemical information for each identified cultivar. Currently, OGDD is designed to enable users to easily retrieve and visualize biologically important information (SSR markers, and olive tree and oil characteristics of about 200 cultivars worldwide) using a set of efficient query interfaces and analysis tools. It can be accessed through a web service from any modern programming language using a simple hypertext transfer protocol call. The web site is implemented in java, JavaScript, PHP, HTML and Apache with all major browsers supported.

**Database URL**: http://www.bioinfo-cbs.org/ogdd/

## Introduction

Olive tree (*Olea europaea* L.) represents the most important oil producing crop in the Mediterranean basin. Its cultivation covers over eight million hectares of land and about 70% of the produced olive oil is consumed. Olive tree is a diploid species (2*n* = 46), whose seeds are produced by cross-pollination (1, 2), is able to survive for a long time. It is a glycophytic species that exhibits a big tolerance to drought and salt stresses when it is compared with other fruit trees that are generally salt sensitive.

Olive oil is the oil extracted from *Olea europaea* L. fruit, using only mechanical methods or other physical procedures that do not alter the glyceric structure of the oil, and therefore conserve its vitamins and other natural healthy high-value compounds. The virgin olive oil is recognized to have beneficial effects on health. In fact, it is able to reduce blood pressure and low-density lipoprotein cholesterol. It has also antioxidant and antimicrobial virtues, such as cancer prevention (3, 4).

According to the Food and Agriculture data from the United Nations, the Mediterranean countries produce 90% of World olives, and the main olive producers are Spain, Italy, Greece, Turkey, Tunisia, Morocco, Syria and Portugal (FAO). Olive and olive oil have great commercial and economical importance in the Mediterranean region. In the last few years, the growth of olive production has expanded throughout the world and olive oil consumption all over the world has significantly increased, and consumers are becoming more informed and increasingly aware of the quality of foods they buy and eat.

Virgin olive oils obtained from one genetic variety of olive or from different varieties are called monovarietal or coupage, respectively. Regarding the monovarietal virgin olive oils, they possess specific characteristics that are associated with the olive variety from which they are extracted. In addition, a high occurrence of mislabeling, homonyms and synonyms has been reported in olive (5). Therefore, it is very important to improve or develop new traceability systems allowing easy and accurate cultivars and oils identification to manage properly the rich variability of olive. A well-documented traceability process and a confirming authenticity tool are needed for the control of the quality of the virgin olive oil introduced in the market. In other words, great effort is being made to obtain a unique and unequivocal genetic profile for every cultivar using molecular markers, since major chemical analyses of virgin olive oils from the similar category but from diverse origins have a limited significance. Indeed, despite the high variability related to the environmental conditions of the different olive groups, their morphological characteristics and the analyses of chemical composition of fatty acid and secondary metabolites are not able to supply reliable results for oil traceability (6–8). For this reason, the genetic identity seems to be the most appropriate technique to identify the variety from which the olive oil under study derives. The use of DNA-based technology in the field of food authenticity, particularly olive oil, is gaining increasing attention.

Recently, the use of molecular markers, such as RAPDs (random amplified polymorphic DNA) (9), AFLPs (amplified fragment length polymorphisms) (10) and SSR (simple sequence repeats), has been recommended to depict virgin olive oil origin and traceability (6, 7, 11, 12). At present, microsatellites (SSRs) are the most relevant genetic markers used in olive cultivar characterization and virgin olive oil authenticity thanks to their numerous properties. Indeed, SSR markers are multiallelic, codominant, highly polymorphic, widely distributed along the plant genomes and easily amenable to PCR-based analyses. Moreover, they have great reproducibility and are currently the most reliable DNA profiling techniques in forensic investigation (13). Indeed, by carrying out Simple Sequence Repeats (SSRs) markers analysis, we can characterize the genetic profile of monovarietal virgin olive oil by comparing the oil-derived pattern with reference database olive oil cultivars. Thus, to efficiently identify and analyse the unknown commercial virgin olive oils, the development of a database including information about olive cultivars and olive oils based on genetic data sets, particularly, SSR markers, becomes necessary. Few online databases have been developed for olive tree (*olea europaea* L.), such as istrian olive database (14), which includes morphological and molecular data of some istrian olive trees and OLEA databases (http://www.oleadb.it/) (15). The latter contain data (microsatellite (SSR) loci) of a wide set of olive cultivars and give the possibility to query for cultivars corresponding to a specific data profile or to look for the variety identity when a profile is available. Another database that can be mentioned is Olea EST database (16). Actually, it is a collection of *Olea europaea* L. and is constructed by first clustering EST reads to produce tentative consensus (TC) sequences and singletons (sESTs). The database annotates and classifies the unique transcripts found according to their biological functions. Other databases such as GLOBAL INVASIVE SPECIES DATABASES (17) remain classical and give simple botanical and biological description of several tree species (such as olea). Among all databases proposed in the literature, only OLEA databases (15) seem to give effective molecular identification of olive cultivars, but they generate only SSR marker size(s) of each olive cultivar without any other information. Therefore, it is necessary to provide public a new available database for identifying an unknown olive tree or oil using SSR markers and providing extended profile description pertaining to the displayed cultivar.

For this purpose, we generated a simple format database of olive species called OGDD, for Olive Genetics Diversity Database, which currently contains many olive tree and oil characteristics (agro-morphological, chemical, genetics (SSR DNA band size(s) …). The created database also integrates a computer application that provides a supplementary test which compares a user-provided SSR fingerprint. In this paper, we present the OGDD database that is currently implemented on the website (http://www.bioinfo-cbs.org/ogdd/) and which can be considered as a reference system in evaluating data obtained from the analysis of unknown samples and in defining the origin and composition of the virgin olive oil. This database can be used by all researchers and stakeholders interested in olive oil field. In the near future, this database will be a useful platform in the traceability and authenticity of olive oil.

## Database construction and data description

### Data extraction

The OGDD site allows anyone interested in the olive oil sector to simply and quickly recover genetic profile from known world-wide olive cultivars. The data included in the ‘OGDD’ derive from publications on morphological, biochemical, sensory and molecular olive oils cultivated world-wide (Table 1). For example, the genetic profiles of different olive oil varieties involved with SSR families are as follows: (DCA (18), GAPU (19), UDO (20), EMO (21), IAS-oli (22, 23), IGP (24). Each family marker is composed of 2 (EMO) to 13 (IAS-oli) markers and for each locus, the allele size of both alleles expressed in base pairs is provided. In the case of homozygosis, both values are equal.

**Table 1.** Data sources of 200 world-wide olive and virgin olive oil cultivars included in the OGDD database

Cultivar origin	Source(s)		
	Agro-morphological data	Pomological and chemical data	Genetic data^a^
**Italy**	(25)	(25, 26, 27, 28, 29)	(25, 30, 31)
**Spain**	(32)	(33)	(31, 34)
**Greece**	(32)	(35, 36)	(31, 34)
**Portugal**	(32)	(37)	(31, 34)
**France**	(38)	(38)	(31)
**Tunisia**	(39)	(36, 39, 40, 41, 42, 43)	(6, 7, 34,44)
**Turkey**	(32)	(45)	(34)
**Syria**	(46)	(46)	(34, 46)
**Morocco**	(32)	(47, 48)	(31, 34, 49)

^a^ Up to now the genetic data existing in the OGDD database are the allele sizes of SSR markers.

Thus, each variety has a range of additional data such as country, region, morphological data represented by four photos (tree, leaf, fruit and kernel), acidity, taste, synonym, bibliographic references, oil content (%), the physico-chemical composition and a matching score calculated to identify each new genetic profile. In fact, a user can compare the genetic profiles of the varieties presented in the database with the genetic profile that s/he experimentally determines. S/He can, therefore, identify the origin of her/his cultivar.

### Data collection and classification

**Figure 1.**
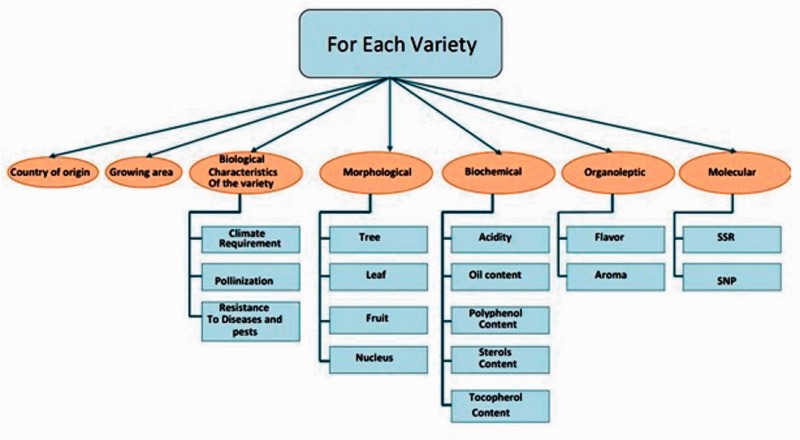
Data classification.

The OGDD data are organized into a database that gathers each type of data separately. The following information is collected for each olive cultivar variety such as the country of origin, the growing region, the biological characteristics of the variety (pollination, climate requirements and resistance to diseases, pests and climates), morphological characteristics (tree, leaf, fruit and nucleus), biochemical characteristics (acidity, oil content, polyphenols, tocopherols, pigments (chlorophylles, carotenes), …), organoleptic characteristics (taste and aroma) and molecular data particularly microsatellite markers (SSR) (Figure 1). For the microsatellite markers, the retrieved data are their name and the groups to which they belong and their allele size (base pairs). OGDD updates are provided manually yearly by the bioinformatics group of the Centre of Biotechnology of Sfax, Tunisia, by checking the SSR markers that are newly obtained by research teams. The current status of the molecular markers data included in OGDD database contains only the SSR markers. In a following step, other molecular markers such as SNP markers will be integrated. Updates include records and complete information concerning *Olea europaea* species.

### Database construction and implementation

OGDD is a browser-independent Web database built using MySQL (version 5.1.41) as the Database Management System. The OGDD database web interface was constructed using HTML, JavaScript and PHP as programming languages. The database was hosted on Apache (version 2.2.14) web server with a Linux operating system. The web tool is compatible with all major browsers including FireFox, Safari, Chrome and Internet Explorer.

**Figure 2.**
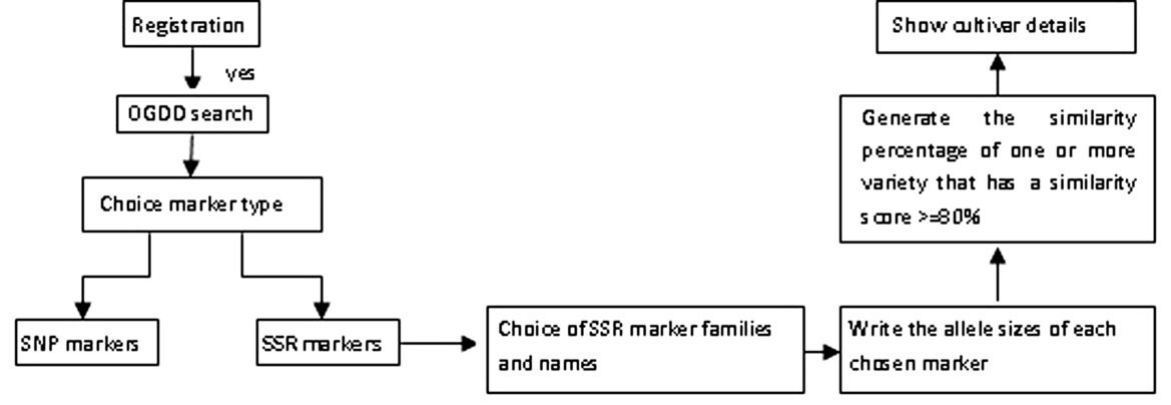
A schematic architecture of OGDD database. Solid arrows mean that the user can choose the analysis path according to results obtained at each previous step. Dashed lines mean that user can return to the OGDD search if he didn’t find the required SSR.

The architecture of OGDD is shown in Figure 2. The data in OGDD can be used to compute the similarity of an unknown virgin olive oil variety based on the SSR information and compare with those existing in the database.

### Database use and access

The major goal for OGDD is to develop an approach toward identification, traceability and authenticity of virgin olive oil to protect the interest of both consumers and producers. OGDD database can be accessed from any computer with web-access, just requiring the registration of the user.

### Algorithm development and score calculating

**Figure 3.**
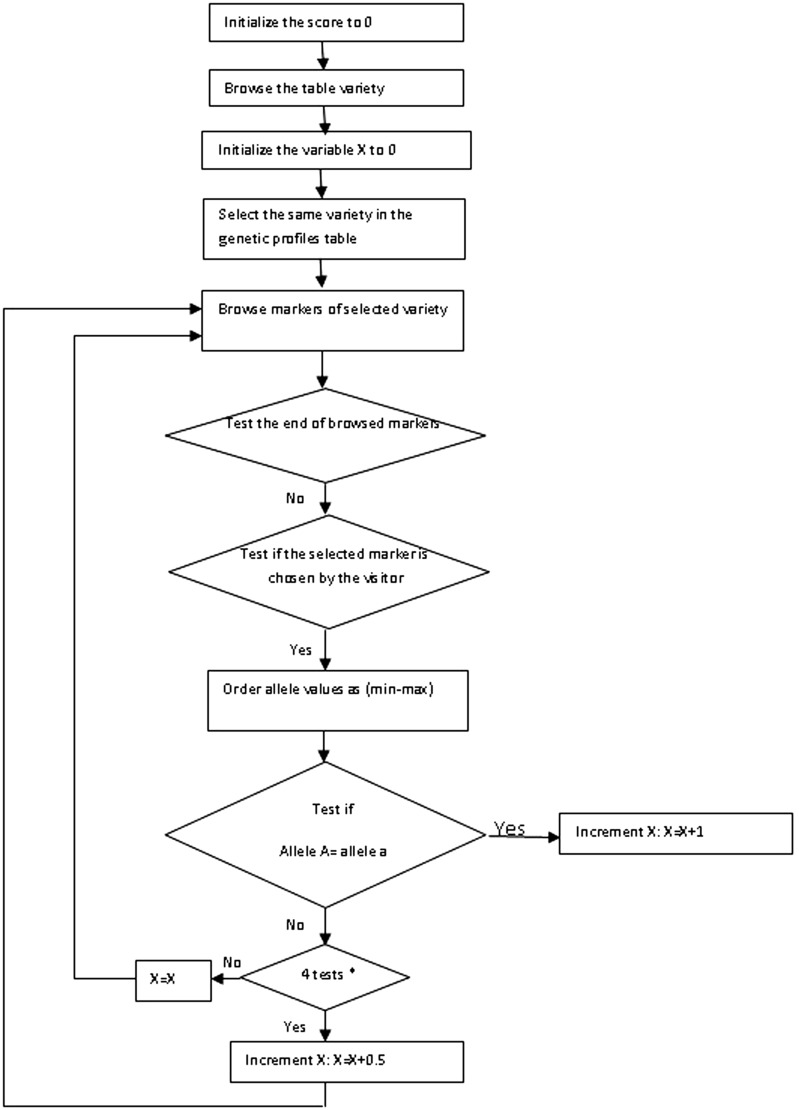
Algorithme structure.

To help the users know and identity the origin of the virgin olive oil genetic profile they have, we propose the diagram of the implemented algorithm that identifies the unknown variety based on the data available in the database by computing the probabilities of homology with the unknown virgin olive oil variety user input (Figure 3). The result is displayed as a percentage of homology.

The graphical depiction of the algorithm is given in Figure 2. The scoring computation will proceed with four major steps. First, it does not only use an intermediate variable *X* for the exact calculating score(s), but also initializes the scores range to zero for all cultivars existing in the present database. Second, for each variety it initializes the variable *X* to zero, selects the genetic profile of every variety and checks all markers of this variety already chosen by the user: if yes, the user must enter the size of the markers of the unknown cultivar. Third, the scoring computation compares allele sizes entered to the database with those of all varieties existing in the OGDD for the same marker. The *X* value is incremented according to the allele sizes of the existing varieties in OGDD. In fact, it is 1 if the two allele sizes are equal, 0.5 when only one allele size is equal and 0 when the two allele sizes are different. Finally, it calculates the score after the comparison of SSR markers. The score by this method depends on both similarities between the allele sizes and the number of SSR markers.

Calculate the score: ***Score = X/N***

$$\begin{array}{l} X:\text{ sum of the elementary scores for each marker}\\ N:\text{ number of markers typed by the user} \end{array}$$

Select the varieties that have a score ≥ 0.8.

If there is no variety that satisfies this condition, the result will be: ‘Not found variety in this genetic profile’.

### OGDD web site interrogation

Once the parameters of the database are defined and the data are grouped, the database is created physically with php / MySQL.

**Figure 4.**
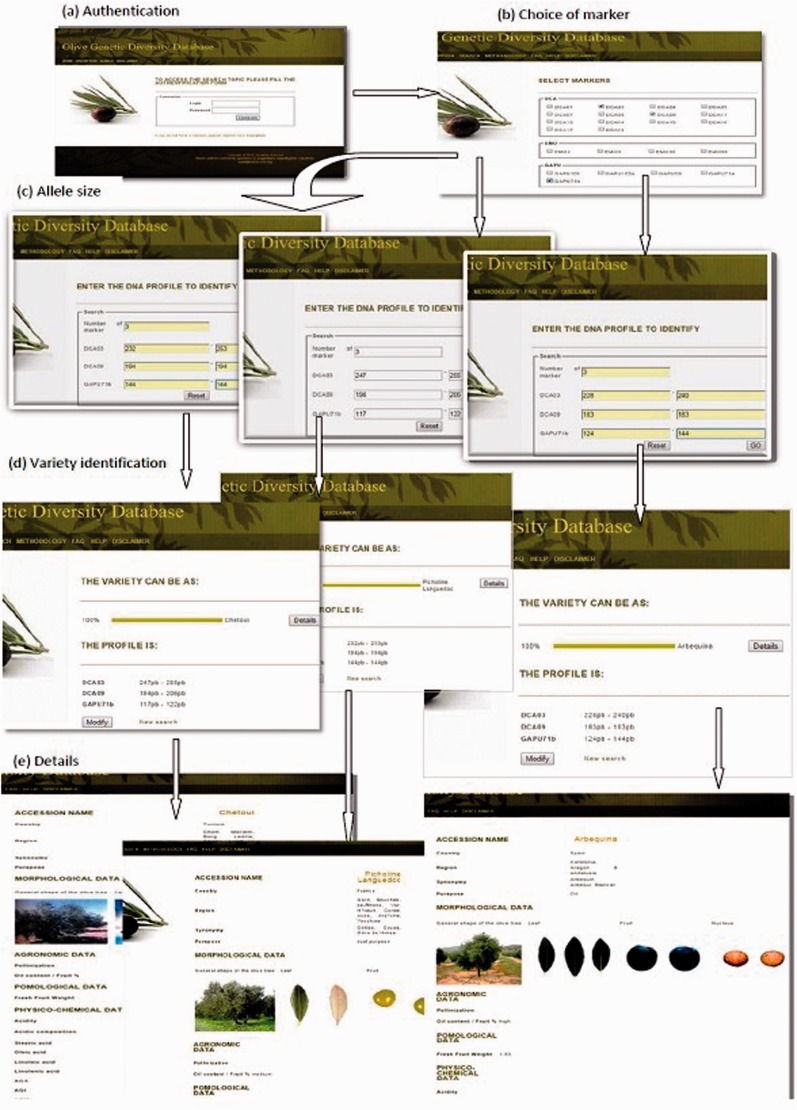
A workflow to identify and visualize a monovarietal olive oil using OGDD database. (**a**) Authentication page where user needs to login, (**b**) Choice of marker page with selection of desired markers, (**c**) Allele size page specifying the size of the allele. (**d**) Variety identification, displaying the resulting monovarietal olive oil cultivar, (**e**) Details: displaying characteristics (morphological, agronomical, physicochemical data) of the identified cultivar.

Querying the database needs a registration for a user account. The administrator has the option to accept or reject the user access permission. In addition, the administrator must authenticate to access the session and perform the functions described in this page. Once accepted, the user must log, check the boxes to select markers, enter the marker numbers and the allele sizes. The result is a homology probability of the unknown variety in question with the varieties of the database. In addition, for each displayed result, we have a link to the details of the variety, namely, the morphology of the tree, leaves and fruit, biochemical characteristics, oil organoleptic quality and allele sizes of all SSR markers existing until now in the database. Such a result is illustrated in Figure 4 following an interrogation case of our database. Finally, the user can edit or modify his/her search.

### Case examples of unknown varieties

Based on the algorithm described above, we have developed software for data basing and managing SSR DNA fingerprint profiles. A query profile can be compared with all fingerprint profiles included in the database, resulting in a profile list formed at decreasing similarity percentages. Only the results with similarity levels higher than 80% are displayed.

Three profile samples (SSR fingerprints of three unknown cultivars) have been separately tested in the OGDD database. The objective was to identify with accuracy these three varieties from their SSR fingerprint profiles. Therefore, for each unknown cultivar, we selected three markers (DCA03, DCA09, GAPU71b). Allele marker sizes were respectively included as follows: 232/253, 194/194, 144/144 for the first sample; 247/255, 194/206, 117/122 for the second sample and 228/240, 183/183, 124/144 for the third sample. For each examined cultivar, the displayed result showed 100% similarity to *Chetoui, Picholine Languedoc* and *Arbequina* cultivar, respectively. Clicking on details, the database displayed characteristics (such as, morphological, agronomical, physicochemical data) for each resulting monovarietal virgin olive oil cultivar (Figure 4).

## Conclusion

OGDD, which is a new and comprehensive database, has been developed focusing on the identification and authentication of olive plant. Compared with other few existing databases for olive species, OGDD has its own specific features and advantages. In fact, it provides a reference system in evaluating the data obtained from the analysis of unknown samples and in defining the varietal composition of the virgin olive oil. It also contains a great deal of information for each cultivar identified by the user. This database can be used by all researchers and stakeholders interested in olive cultivar identification and virgin olive oil authentication. Up to now, OGDD initializes the search only by SSR markers. However, its next update extends the query by adding other molecular markers, such as, SNP and includes more information of other olive cultivars.

## References

[1] Doveri S. Donal M. Lee D. (2006) Non concordance between genetics profiles of olive oil and fruit: a cautionary note to the use of DNA markers for provenance testing. J. Agric. Food Chem., 54, 9221–9226. 17117813 10.1021/jf061564a

[2] Besnard G. Khadari B. Villemur P. (2000) Cytoplasmic male sterility in the olive (*Olea europaea* L.). Theor. Appl. Genet., 100, 1018–1024.

[3] Elloumi J. Ben-Ayed R. Aifa S. (2012) An overview of olive oil biomolecules. Curr. Biotechnol., 1, 115–124.

[4] Owen R. Giacosa A. Hull E. (2000) Olive-oil consumption and health: the possible role of antioxidants. Lancet Oncol., 1, 107–121. 11905662 10.1016/s1470-2045(00)00015-2

[5] Barranco D. Rallo L. (2000) Olive cultivars in Spain. HortTechnology, 10, 107–110.

[6] Ben-Ayed R. Grati-Kamoun N. Moreau F. (2009) Comparative study of microsatellite profiles of DNA from oil and leaves of two Tunisian olive cultivars. Eur. Food Res. Technol., 229, 757–762.

[7] Ben-Ayed R. Grati-Kamoun N. Sans-grout C. (2012) Characterization and authenticity of virgin olive oil (*Olea europaea* L.) cultivars by microsatellite markers. Eur. Food Res. Technol., 234, 263–271.

[8] Ben-Ayed R. Grati-Kamoun N. Rebai A. (2013) An overview of the authentication of olive tree and oil. Compr. Rev. Food Sci. F., 12, 218–227.

[9] Busconi M. Foroni C. Corradi M. (2003) DNA extraction from olive oil and its use in the identification of the production cultivar. Food Chem., 83, 127–134.

[10] Pafundo S. Agrimonti C. Marmiroli N. (2005) Traceability of plant contribution in olive oil by amplified fragment length polymorphisms. J. Agric. Food Chem., 53, 6995–7002. 16131101 10.1021/jf050775x

[11] Testolin R. Lain O. (2005) DNA extraction from olive oil and PCR amplification of microsatellite markers. Food Sci., 70, 108–112.

[12] Ben-Ayed R. Sans-grout C. Moreau F. (2014) Genetic similarity among Tunisian olive cultivars and two unknown feral olive trees estimated through SSR markers. Biochem. Genet., 52, 258–268. 24535154 10.1007/s10528-014-9645-x

[13] Jobling M.A. Gill P. (2004) Encoded evidence: DNA in forensic analysis. Nat. Rev. Genet., 5, 739–751. 15510165 10.1038/nrg1455

[14] Istrian Olive Database (2005) http://www.iptpo.hr/iod/

[15] Olea databases (2008) http://www.oleadb.it/

[16] Olea EST database (2010) http://140.164.45.140/oleaestdb/

[17] GLOBAL INVASIVE SPECIES DATABASES: http://www.issg.org/database/welcome/

[18] Sefc K.M. Lopes M.S. Mendoncxa D. (2000) Identification of microsatellite loci in olive (*Olea europaea*) and their characterization in Italian and Iberian olive trees. Mol. Ecol., 9, 1171–1173. 10964237 10.1046/j.1365-294x.2000.00954.x

[19] Carriero F. Fontanazza G. Cellini F. (2002) Identification of simple sequence repeats (SSRs) in olive (*Olea europaea* L.). Theor. Appl. Genet., 104, 301–307. 12582701 10.1007/s001220100691

[20] Cipriani G. Marrazzo M.T. Marconi R. (2002) Microsatellite markers isolated in olive (*Olea europaea* L.) are suitable for individual fingerprinting and reveal polymorphism within ancient cultivars. Theor. Appl. Genet., 104, 223–228. 12582690 10.1007/s001220100685

[21] De La Rosa R. James C.M. Tobutt K.R. (2002) Isolation and characterization of polymorphic microsatellites in olive (*Olea europaea* L.) and their transferability to other genera in the Oleaceae. Mol. Ecol. Notes, 2, 265–267.

[22] Diaz A. Martin A. Rallo P. (2006) Self-incompatibility of ‘Arbequina’ and ‘Picual’ olive assessed by SSR markers. J. Am. Soc. Hortic. Sci., 131, 250–255.

[23] Rallo P. Dorado G. Martin A. (2000) Development of simple sequence repeats (SSRs) in olive tree (*Olea europaea* L.). Theor. Appl. Genet., 101, 984–989.

[24] Busconi M. Sebastiani L. Corrado F. (2006) Development of SCAR markers for germplasm characterisation in olive tree (*Olea europaea* L.). Mol. Breed., 17, 59–68.

[25] Muzzalupo I. **(**2012) Olive germplasm—Italian catalogue of olive varieties. http://dx.doi.org/10.5772/3577.

[26] Brescia M.A. Alviti G. Liuzzi V. (2003) Chemometrics classification of olive cultivars based on compositional data of oils. J. Am. Oil Chem. Soc., 80, 945–950.

[27] Mannina L. Dugo G. Salvo F. (2003) Study of the cultivar-composition relationship in Sicilian olive oils by GC, NMR, and statistical methods. J. Agric. Food Chem., 51, 120–127. 12502395 10.1021/jf025656l

[28] Nagy K. Bongiorno D. Avellone G. (2005) High-performance liquid chromatography-mass spectrometry-based chemometric characterization of olive oils. J. Chromatogr. A, 1078, 90–97. 16007986 10.1016/j.chroma.2005.05.008

[29] Cerretani L. Bendini A. Del Caro A. (2006) Preliminary characterisation of virgin olive oils obtained from different cultivars in Sardinia. Eur. Food Res. Technol., 222, 354–361.

[30] Muzzalupo I. Stefanizzi F. Perri E. (2009) Evaluation of olives cultivated in southern Italy by simple sequence repeat markers. HortScience, 44, 582–588.

[31] Doveri S. Sabino Gil F. Díaz A. (2008) Standardization of a set of microsatellite markers for use in cultivar identification studies in olive (*Olea europaea* L.). Sci. Hortic., 116, 367–373.

[32] World Catalogue of Olive Varieties (International Olive Council, 2000).

[33] Garcia A. Brenes M. Garcia P. (2003) Phenolic content of commercial olive oils. *Eur**. *Food Res. Technol., 216, 520–525.

[34] Diaz A. Martin A. Rallo P. (2006) Self-incompatibility of ‘Arbequina’ and ‘Picual’ olive assessed by SSR markers. J. Am. Soc. Hort. Sci., 131, 250–255.

[35] Alves M.R. Cunha S.C. Amaral J.S. (2005) Classification of PDO olive oils on the basis of their sterol composition by multivariate analysis. Anal. Chim. Acta, 549, 166–178.

[36] Manai H. Mahjoub-Haddada F. Oueslati I. (2008) Characterization of monovarietal virgin olive oils from six crossing varieties. Sci. Hortic., 115, 252–260.

[37] Cunha S.C. Amaral J.S. Fernandes J.O. (2006) Quantification of tocopherols and tocotrienols in Portuguese olive oils using HPLC with three different detection systems. J. Agric. Food Chem., 54, 3351–3356. 16637695 10.1021/jf053102n

[38] Moutier N. Pinatel C. Martre A. (2004) Identification et caractérisation des variétés d'olivier cultivées en France (tome1). Naturalia Publications, Turriers, p. 248.

[39] Khlif M. Grati N. (2001) Caractérisation technologique des variétés d’olivier cultivées en Tunisie. Ezzaitouna ISSN: 0330-6828.

[40] Issaoui M. Flamini G. Brahmi F. (2010) Effect of the growing area conditions on differentiation between Chemlali and Chétoui olive oils. Food Chem., 119, 220–225.

[41] Baccouri O. Cerretani L. Bendini A. (2007) Preliminary chemical characterization of Tunisian monovarietal virgin olive oils and comparison with Sicilian ones. Eur. J. Lipid Sci. Technol., 109, 1208–1217.

[42] Guerfel M. Ben Mansour M. Ouni Y. (2012) Triacylglycerols composition and volatile compounds of virgin olive oil from Chemlali cultivar: comparison among different planting densities. SciWorld J., 2012, ID 354019. 10.1100/2012/354019 PMC3353306 22629139

[43] Tena N. Lazzez A. Aparicio-Ruiz R. (2007) Volatile compounds characterizing Tunisian Chemlali and Chétoui virgin olive oils. J. Agric. Food Chem., 55, 7852–7858. 17708651 10.1021/jf071030p

[44] Taamalli W. Geuna F. Banfi R. (2006) Agronomic and molecular analyses for the characterisation of accessions in Tunisian olive germplasm collections. J. Biotechnol., 9, 467–81.

[45] Dıraman H. (2010) Characterization by chemometry of the most important domestic and foreign olive cultivars from the National Olive Collection Orchard of Turkey. Grasas Aceites, 61, 341–351.

[46] Jibara G. Ashtar S. Jawhar A. (2007) Oil quality and morphological, phenological, bio-agronomical and molecular characterization of Syrian *Olea europaea* L. germplasm. In: Di Terlizzi B. Dragotta A. Jamal M. (eds.). Syrian national strategic plan for olive oil quality: final report. Bari* : **CIHEAM.* pp. 85–94.

[47] Essiari M. Zouhair R. Chimi H. (2014) Contribution to the study of the typical characteristics of the virgin olive oils produced in the region of Sais (Morocco). Olivae, 119, 8–21.

[48] Haddam M. Chimi H. El-Antari A. (2014) Physico-chemical characterisation and oxidative stability of olive oils produced from the ‘Picholine marocaine', ‘Haouzia', ‘Koroneiki' and ‘Arbequina' varieties in the central olive growing region of Morocco (Chaouia-Ouardigha) Olivae, 119, 22–34.

[49] Khadari B. Charafi J. Moukhli A. (2008) Substantial genetic diversity in cultivated Moroccan olive despite a single major cultivar: a paradoxical situation evidenced by the use of SSR loci. Tree Genet. Genomes, 4, 213–221.

